# Adenosine and P1 receptors: Key targets in the regulation of sleep, torpor, and hibernation

**DOI:** 10.3389/fphar.2023.1098976

**Published:** 2023-03-10

**Authors:** Wei-Xiang Ma, Ping-Chuan Yuan, Hui Zhang, Ling-Xi Kong, Michael Lazarus, Wei-Min Qu, Yi-Qun Wang, Zhi-Li Huang

**Affiliations:** ^1^ State Key Laboratory of Medical Neurobiology, MOE Frontiers Center for Brain Science, Department of Pharmacology, School of Basic Medical Sciences, Institutes of Brain Science, Fudan University, Shanghai, China; ^2^ Anhui Provincial Engineering Research Center for Polysaccharide Drugs, Provincial Engineering Laboratory for Screening and Re-evaluation of Active Compounds of Herbal Medicines in Southern Anhui, School of Pharmacy, Wannan Medical College, Wuhu, China; ^3^ International Institute for Integrative Sleep Medicine (WPI-IIIS) and Faculty of Medicine, University of Tsukuba, Tsukuba, Ibaraki, Japan

**Keywords:** adenosine, P1 receptors, hibernation, sleep, torpor

## Abstract

Sleep, torpor, and hibernation are three distinct hypometabolic states. However, they have some similar physiological features, such as decreased core body temperature and slowing heart rate. In addition, the accumulation of adenosine seems to be a common feature before entry into these three states, suggesting that adenosine and its receptors, also known as P1 receptors, may mediate the initiation and maintenance of these states. This review, therefore, summarizes the current research on the roles and possible neurobiological mechanisms of adenosine and P1 receptors in sleep, torpor, and hibernation. Understanding these aspects will give us better prospects in sleep disorders, therapeutic hypothermia, and aerospace medicine.

## Graphical Abstract

**Figure:**
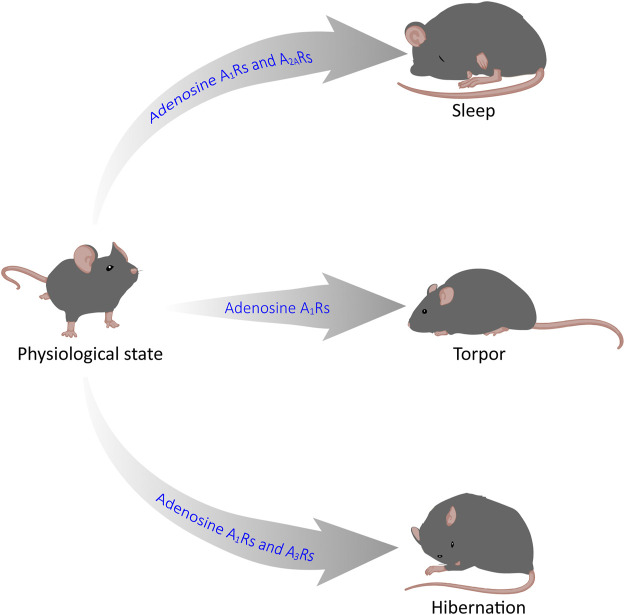
Adenosine mediates sleep, torpor and hibernation through P1 receptors. Recent reasearch has shown that P1 receptors play a vital role in the regulation of sleep-wake, torpor and hibernation-like states. In this review, we focus on the roles and neurobiological mechanisms of the CNS adenosine and P1 receptors in these three states. Among them, A_1_ and A_2A_ receptors are key targets for sleep-wake regulation, A_1_Rs and A_3_Rs are very important for torpor induction, and activation of A_1_Rs is sufficient for hibernation-like state.

## 1 Introduction

Sleep, torpor, and hibernation are three distinct states which can reduce energy expenditure. Sleep, which takes up nearly one-third lifetime of most mammals and birds, is divided into rapid eye movement (REM) sleep and non-REM (NREM) sleep. REM sleep is characterized by phasic changes in various autonomic functions and an elevation in metabolic rate. However, NREM sleep is characterized by the organism’s active contact with the environment and by a decrease in metabolism, body temperature (T_b_), and energy expenditure (Silvani and Dampney, 2013; Schmidt, 2014; Silvani et al., 2018). Torpor is an energy-saving strategy in most mammals and birds, sometimes lasting only for a few hours, that helps organisms cope with the stress of an adverse environment (Ruf and Geiser, 2015). Just like NREM sleep, torpor state occurs with a reduction in T_b_ and metabolic rate (Ruf and Geiser, 2015). Hibernation, also called multi-day torpor, is a seasonal energy conservation strategy that reduces T_b_, energy expenditure, and water loss (Geiser, 2013; Ruf and Geiser, 2015). Most hibernators generally remain in hibernation for a winter, which helps them effectively withstand the cold environment.

Adenosine is a ubiquitous endogenous cell signal transducer and regulator, which mainly acts by activating 4 G protein-coupled receptors (GPCRs), namely, adenosine A_1_, A_2A_, A_2B,_ and A_3_ receptors, as known as P1 receptors (Kazemzadeh-Narbat et al., 2015). Activation of A_1_ and A_3_ receptors exert inhibitory effects, however A_2A_ and A_2B_ exert excitatory. The four P1 receptors can reduce and increase the intracellular cyclic adenosine-3, 5 monophosphate (cAMP) concentration *via* inhibiting or activating adenylate cyclase (AC), which makes adenosine and P1 receptors essential for the regulation of energy balance (Chiu and Freund, 2014).

Sleep, torpor, and hibernation are integral to energy balance. At the same time, adenosine which is a homeostatic bioenergetic network regulator appears to accumulate before entry into the three states, suggesting that adenosine and P1 receptors, may mediate sleep, torpor and hibernation (Drew and Jinka, 2013; Silvani et al., 2018). Much evidence suggests that activation or inhibition of the central nervous system (CNS) adenosine receptors by genetic or pharmacological means can alter the states of sleep, torpor, and hibernation. In this review, we focus on the role of adenosine in the CNS and summarize the current research on the roles and possible biological mechanisms of adenosine and P1 receptors in sleep, torpor, and hibernation. This may help us solve many problems in the future, such as treating sleep disorders and using artificial hibernation for medical applications and space exploration.

## 2 Physiological characteristics during sleep, torpor, and hibernation

Sleep, torpor, and hibernation appear shallow to deep states of diminished body temperature and metabolic rate. Sleep is a relatively rapid and reversible state. However, the animals in a torpor state are more difficult to awaken than sleepers. They may not respond immediately to stimuli, while hibernators typically take an hour or more from hibernation to awakening (Siegel, 2009). Animals control the duration of torpor based on the circadian system, typically remaining dormant for only part of the day and returning to a physiological state when T_b_ rises to a consistently high level.

In contrast to torpor, hibernation lasts for days or weeks, and hibernators generally do not forage, relying mainly on early food storage or fat storage (Ruf and Geiser, 2015). Hibernation is not as common as daily sleep and torpor; only one-third of mammalian species are hibernators (Berger, 1984). Sleep, torpor, and hibernation are both energy-saving strategies for animals that share similar physiological characteristics and have their own characteristics (Table 1). An interesting commonality between sleep, torpor, and hibernation is the involvement of adenosine receptors. Adenosine is a purine nucleoside involved in many signaling pathways of energy homeostasis. One of the functions of sleep is to restore brain energy homeostasis, while the primary function of hibernation and torpor is to restore or protect body energy homeostasis (Drew and Jinka, 2013). According to many previous studies, adenosine A_1_ receptors and A_2A_ receptors (A_1_Rs and A_2A_Rs) play an essential role in inducing NREM, the activation of A_1_R and A_3_ receptors (A_3_Rs) may induce torpor (Silvani et al., 2018), and the onset of hibernation may be due to the activation of A_1_Rs (Jinka et al., 2011; Frare and Drew, 2021). In the following, we will briefly introduce the physiological characteristics of the three states and expand our review based on this.

**TABLE 1:** Physiological characteristics of sleep, daily torpor, and hibernation.

	Sleep	Torpor	Hibernation	References
Energy saving	5%–15%	60%–70%	>90%	Swoap et al. (2017), Mohr et al. (2020)
Metabolic rate	70%–90% of BMR	∼35% of BMR	6% of BMR	Ruf and Geiser (2015)
BP (relative decrease to normal value)	∼10%	25%–30%	40%–80%	Silvani and Dampney (2013), Ambler et al. (2021)
Body temperature (the decrease compared to 36°C–40°C)	<3°C	5°C–20°C	15°C–35°C	Berger (1984)
Respiration rate (% of active state)	100%–80%	5%–20%	2%–3%	Mohr et al. (2020)
HR (% of active state)	70%–90%	10%–30% minimum HR (70 to 150 bpm)	1%–4% minimum HR (5 to 10 bpm)	Swoap et al. (2017), Mohr et al. (2020)
EEG (NREM)	↓	↓↓	↓↓↓	Huang et al. (2021)
EMG (NREM)	↓	↓↓	↓↓↓	Huang et al. (2021)
HP	↑	↑	↑	Silvani and Dampney (2013)

Note: ↓: decrease, ↑: increase.

### 2.1 Sleep

Most mammals and birds spend about one-third of their lives asleep, a quiet state in which humans or animals are less sensitive to their environment. Sleep is regulated by biological rhythms and neural loops and plays a vital role in the human body’s functional recovery, learning and memory, and growth and development. It is characterized by loss of consciousness, decreased T_b_, metabolism, and a decrease in heart rate (HR) and blood pressure (BP). According to the characteristic electroencephalographic (EEG) patterns, sleep can be divided into NREM and REM sleep.

NREM and REM sleep occur alternately throughout sleep time, with NREM accounting for the majority of the sleep time (Silvani and Dampney, 2013; Schmidt, 2014; Silvani et al., 2018). NREM sleep shows decreased systemic function, regular breathing, HR, reduced energy consumption, an EEG that consisted mainly of slow waves, reduced muscle tension, but still a definite posture, with no noticeable eye changes. NREM sleep is divided into four stages. Stages Ⅰ and Ⅱ are light sleep, and stages Ⅲ and Ⅳ are deep sleep. During deep sleep, cellular metabolism can be promoted throughout the body, immunity can be strengthened, and energy depleted during the wake period can be restored (Silvani et al., 2018). REM sleep is characterized by rapid eye movement, loss of thermoregulation, EEG activity similar to waking, marked decrease or disappearance of muscle tension, muscle relaxation, but active neurons in most brain regions, increased cerebral blood flow, irregular breathing, and increased HR. During REM sleep, humans or animals maintain a relatively high level of vigilance, which is essential for animals to survive in nature (Roth, 2004; Schmidt, 2014).

### 2.2 Torpor

Torpor, a behavior that saves energy by reducing metabolic rate (MR), is often identical to sleep, which occurs daily or lasts for days, transitions into sleep (also called daily torpor), and is regulated by circadian rhythms (Berger, 1984). A drastic reduction of MR associated with a decrease in T_b_ results in the occurrence of torpor (Giroud et al., 2020). In addition, the autonomic nervous system is intimately involved in all stages of torpor. During an episode of torpor, the respiratory rate decreased, the HR related to ventilation increased periodically, and the decrease in ventilation was more significant than the MR, resulting in mild respiratory acidosis (Silvani et al., 2018).

A decrease in brain temperature usually accompanies the onset of torpor. If the brain temperature is above 25°C, EEG morphology and frequency during torpor are closest to the characteristics of NREM sleep. Then, both EEG amplitude and power decrease with decreasing T_b_. When the brain temperature falls below 25°C, REM sleep gradually disappears, and when the temperature is between 10°C and 20°C, the animals alternate between long NREM sleep and short wakefulness. EEG becomes equipotential when the brain temperature is below 10°C, and it is impossible to determine alertness by electrophysiological methods (Ruf and Geiser, 2015; Ambler et al., 2021; Huang et al., 2021). When electromyography (EMG) was examined, EMG activity was found to decrease significantly with the inhibition of shivering thermogenesis, and a decrease of T_b_ when entering the state of torpor was observed (Huang et al., 2021). Daily torpor appears independent of ambient temperature (Ta), season, and nutritional status, as it can last only a few hours and is frequently interrupted by activity and foraging. Torpor can occur throughout the year, although it is more frequent in winter. However, in some species that live in warm climates, summer torpor is more common than winter torpor. Compared with waking, the metabolic rate drops to an average of about 30% of the basal metabolic rate (BMR) during torpor. The energy consumption is usually reduced by 10% to 80%, depending on the time and depth of torpor (Geiser, 2013).

### 2.3 Hibernation

Hibernation is a physiological adaptation that allows endothermic animals to cope with periodic limitations in their energy supply by lowering T_b_ and metabolism and improve their freezing tolerance, which may enable them to survive seasonal changes in the food supply and temperature reduction (Geiser, 2013; Storey and Storey, 2013; Storey and Storey, 2017). When the metabolic rate decreases during hibernation, ventilation decreases, and prolonged apnea occurs (Milsom and Jackson, 2011). During deep hibernation, the T_b_ of most mammals is near Ta. However, as T_b_ approaches the freezing, MR rises sharply, preventing tissue damage from increased heat production (Milsom and Jackson, 2011; Geiser, 2013). Hibernating species include facultative hibernators (hamsters, bats) and obligatory hibernators (ground squirrels, bears, and lemurs). Facultative hibernators are animals that go into hibernation only when they sense cold, lack of food, or photoperiodic changes. Obligatory hibernators are animals that go into hibernation spontaneously and punctually at a specific time of year, regardless of food availability or temperature (Xu et al., 2013; Mohr et al., 2020).

Hibernation is not an uninterrupted process over several months. With the rise of Ta and the accumulation of metabolites, spontaneous periodic awakening may occur and interrupt dormancy. After a brief awakening, the animal returns to dormancy and repeats the cycle of dormancy-awakening until the end of hibernation. This periodic awakening consumes most of the energy during hibernation. The onset of hibernation is highly dependent on temperature. When Ta is between 20°C and 30°C, some species still hibernate, but the duration is usually only a few hours, similar to daily torpor (Geiser, 2013; Ruf and Geiser, 2015; Mohr et al., 2020; Ambler et al., 2021). Gene transcription and translation are significantly inhibited during hibernation, and many other physiological parameters are significantly reduced and recover after awakening, such as HR, respiration, metabolic rate, and so on (Xu et al., 2013).

## 3 Sources and metabolic pathways of adenosine in the central nervous system

### 3.1 Source of adenosine

**FIGURE 1:**
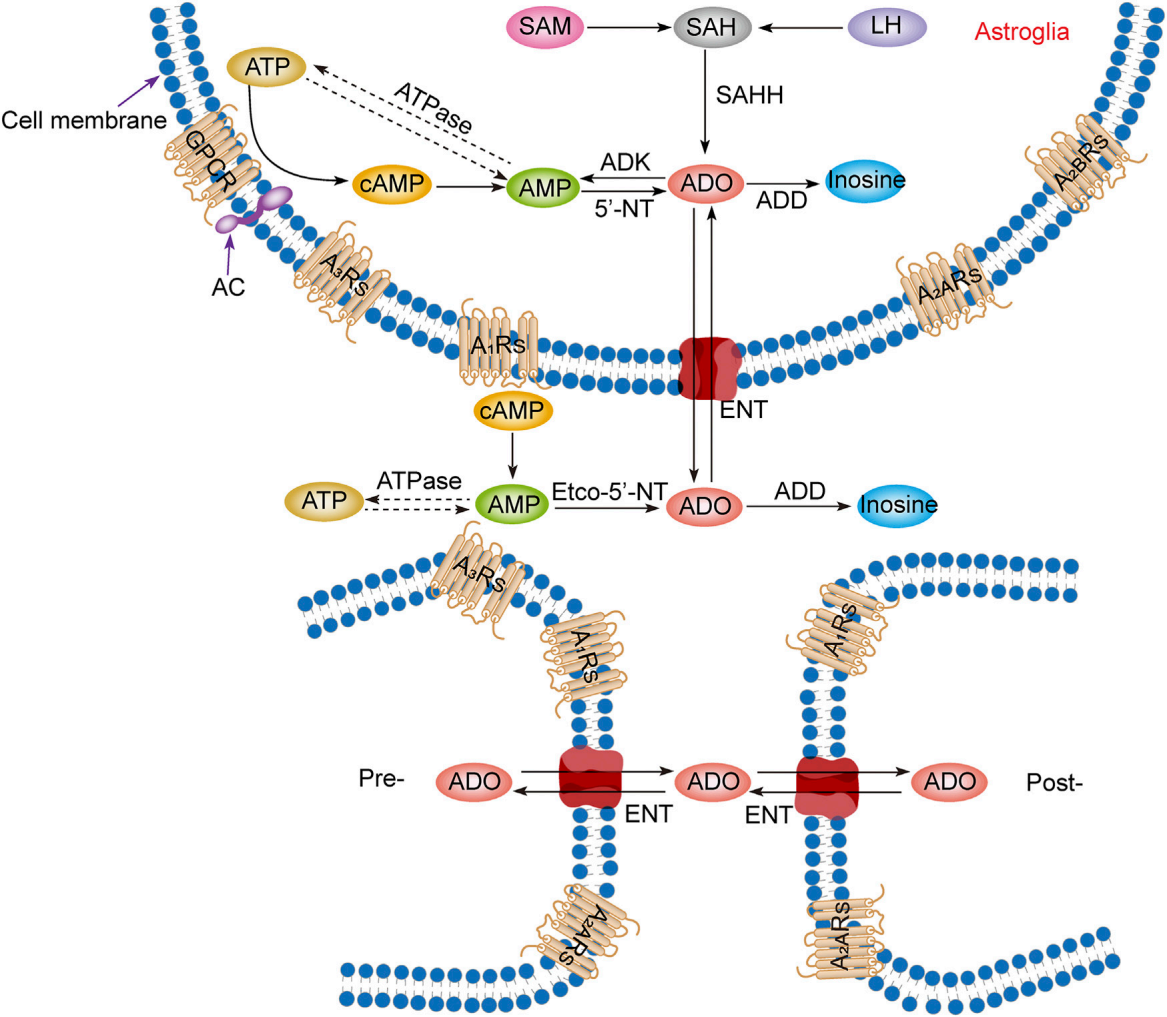
Adenosine metabolism and P1 receptors in the central nervous system. Adenosine metabolism occurs mainly in neuronal synapses and astrocytes. In cells, adenosine is formed from ATP, cAMP, or SAH. Extracellular adenosine is produced by ATP and cAMP metabolism but mainly by the balance of nucleoside transporters to regulate the concentration level inside and outside the membrane. SAM, S-adenosylmethionine; SAH, S-adenosyl homocysteine; LH, L-homocysteine; SAHH, S-adenosyl homocysteine hydrolase; ATP, adenosine triphosphate; ADP, adenosine diphosphate; AMP, adenosine monophosphate; cAMP, cyclic adenosine monophosphate; ADO, adenosine; ADD, adenosine deaminase; ADK, adenosine kinase; 5′-NT, 5′-nucleotidase; etco-5′-NT, etco-5′-nucleotidase; AC, adenylate cyclase; GPCR, G protein-coupled receptors; ENT, equilibrating nucleoside transporter; A_1_Rs, adenosine A_1_ receptors; A_2A_Rs, adenosine A_2A_ receptors; A_2B_Rs, adenosine A_2B_ receptors; A_3_Rs, adenosine A_3_ receptors; Pre-, presynaptic membrane; Post-, postsynaptic membrane.

Intracellular adenosine is mainly produced through five pathways (Figure 1): 1) Adenosine triphosphate (ATP) loses two phosphate groups under the action of ATPase to become adenosine monophosphate (AMP), and AMP continues to lose the phosphate group under the action of an internal 5′-nucleotidase (5′-NT) to produce adenosine (Lopes et al., 2011). 2) Adenine reacts with 1-phosphate ribose to form adenosine (Hall and Frenguelli, 2018). 3) S-adenosylmethionine (SAM) and L-homocysteine produce S-adenosylhomocysteine (SAH) and further produce adenosine under the action of S-adenosylhomocysteine hydrolase (SAHH), but this pathway is not common in the CNS (Deussen et al., 1989; Latini and Pedata, 2001). 4) Extracellular adenosine is transported into the cell by the balanced nucleoside transporter in the cell membrane (Liu Y. J. et al., 2019). 5) cAMP is generated from ATP under the action of AC, which is regulated by GPCRs, and then converted through phosphodiesterases (PDEs) to AMP, which is eventually used to generate adenosine (Dos Santos-Rodrigues et al., 2015).

Production of extracellular adenosine occurs mainly by two pathways (Figure 1): 1) intracellular adenosine is transported to the extracellular space by the balanced nucleoside transporter located in the cell membrane (Sala-Newby et al., 1999). 2) Extracellular ATP and adenosine diphosphate (ADP) are converted to AMP by the enzyme ecto-nucleoside triphosphate diphosphohydrolase (E-NTPDase), also known as CD39. Subsequently, adenosine is generated by ecto-5′-nucleotidase (ecto-5′-NT), also known as CD73, which is mainly expressed on astrocytes, oligodendrocytes and microglia (Lazarus et al., 2019a).

In the equilibrium state, the intracellular adenosine level is 100 nM, and the extracellular adenosine level is 140–200 nM (Dunwiddie and Diao, 1994), but in the pathological state, such as ischemia and hypoxia, extracellular adenosine level increases three- to 10-fold (Andiné et al., 1990; Dux et al., 1990). It is worth noting that although adenosine can be produced from the synaptic terminals of neurons and enter the synaptic space, it is not secreted through vesicles but transported through nucleoside transporters, which has nothing to do with neural activities. Thus, adenosine is not a neurotransmitter but a regulatory factor (Huang et al., 2011; Lopes et al., 2011; Huang et al., 2014).

### 3.2 Adenosine metabolism

Adenosine has three main metabolic pathways (Figure 1): 1) It becomes inosine under the action of adenosine deaminase [8], and then generates hypoxanthine and hypoxanthine nucleotides by nucleoside phosphorylase, and finally becomes uric acid (Fredholm et al., 2005). 2) Adenosine is transported intracellular and extracellular domain through two-way balanced nucleoside transporter to regulate intracellular and extracellular adenosine levels (Liu Y. J. et al., 2019). 3) Adenosine kinase (ADK), which is mainly found in astrocytes, generates AMP and ADP by phosphorylating adenosine in the presence of ATP. This metabolic pathway can only occur in cells, so extracellular adenosine must enter cells to complete the cycle (Huang et al., 2011; Huang et al., 2014; Garcia-Gil et al., 2021).

## 4 Excitatory and inhibitory effects of various adenosine receptors

The physiological functions of adenosine is mediated by four purinergic type 1 receptors, known as A_1_, A_2A_, A_2B,_ and A_3_ receptors, which belong to GPCR family. A_1_Rs and A_3_Rs belong to the inhibitory adenylate cyclase G protein (Gi) family, whereas A_2A_Rs and A_2B_Rs belong to the stimulatory adenylate cyclase G protein (Gs) family (Wall and Dale, 2008; Lopes et al., 2011).

### 4.1 A_1_ receptors

A_1_Rs have the highest affinity for adenosine and can be activated when the concentration of adenosine is in the pM range. They are the most prominent adenosine receptor in the CNS, distributed mainly in the cerebral cortex, hippocampus, and thalamus. A_1_Rs are located primarily in the excitatory nerve terminals (Kashfi et al., 2017). Activation of A_1_Rs can inhibit the activity of adenylate cyclase (AC), decrease the cAMP content, and regulate the activity of cAMP-dependent protein kinase. A_1_R activation can increase the release of intracellular Ca^2+^, inhibit N-, Q- and P-type calcium channels, decrease the influx of extracellular Ca^2+^, block the release of neurotransmitters, and reduce neuronal discharge to regulate neuronal activity (Wall and Dale, 2008). In the postsynaptic membrane, A_1_Rs are activated to open K^+^ channels and increase K^+^ outflow, resulting in membrane hyperpolarization, which reduces excitability and protects neurons. When activated, A1Rs can also open the ATP-sensitive potassium channel (KATP) of substantia nigra neurons, increasing outward currents and decreasing membrane excitability (Stockwell et al., 2017).

### 4.2 A_2A_ receptors

The affinity of A_2A_Rs for adenosine is lower than that of A_1_Rs, and the activation concentration of adenosine is in the nM range. A_2A_Rs are mainly distributed in dopaminergic areas, such as striatum, nucleus accumbens (NAc), olfactory nodules and so on (Fang et al., 2017; Dong et al., 2022). When A_2A_Rs are activated, they are coupled with Gs protein in the brain to increase the activity of AC and cAMP in striatal cells. In the hippocampus, A_2A_Rs appear to be coupled with Gi/Go protein (Diógenes et al., 2004). A_2A_Rs are mainly expressed in D_2_ dopamine receptor cells and are particularly abundant in the plasma membrane of dendrites and dendritic spines, but less so in axons, axon terminals, and glial cells, and has an antagonistic effect with dopamine D_2_ receptors (D_2_Rs) (Ferre et al., 1991; Strömberg et al., 2000). Presynaptic A_2A_Rs can regulate the inhibition of A_1_Rs. In contrast to A_1_Rs, adenosine promotes the release of excitatory transmitters by activating A_2A_Rs. In astrocytes, A_2A_Rs are involved in the regulation of glutamate release and γ-aminobutyric acid (GABA) uptake (Cristóvão-Ferreira et al., 2009). The balance between A_1_ and A_2A_Rs is crucial to the adenosine response, and this close interaction between them can produce a response that is different from the sum of the two (Chiu and Freund, 2014).

### 4.3 A_2B_ receptors

A_2B_Rs have a low affinity for adenosine, and the activation concentration of adenosine should reach μM, suggesting that A_2B_Rs mainly play a role under pathological conditions with increased extracellular adenosine concentration. A_2B_Rs are primarily distributed in hippocampal neurons and glial cells, and a small amount is also found in the thalamus, lateral ventricle, and striatum. A_2B_Rs can activate AC *via* Gs or phospholipase C (PLC) *via* Gq. Activation of A_2B_Rs can increase intracellular cAMP, promote glycogen decomposition, and increase the energy supply of neurons to resist the pathological state of ischemia and hypoxia (van Calker et al., 1979; Hösli and Hösli, 1988; Dos Santos-Rodrigues et al., 2015).

### 4.4 A_3_ receptors

A_3_Rs have the lowest sensitivity compared to other adenosine receptors, but activation of A_3_Rs has neuroprotective and neurotrophic effects. Although A_3_Rs are distributed throughout the brain, their content varies greatly in different brain regions, especially in the hippocampus and cerebellum. A_3_Rs act through Gi-mediated AC inhibition and Gq-mediated PLC activation. A_3_Rs can regulate hippocampal synaptic plasticity and decrease adenylate cyclase activity. In short, A_3_Rs activation is closely related to inflammation inhibition and cell protection (Lopes et al., 2003; Vlajkovic et al., 2007; Lopes et al., 2011).

## 5 The roles and neurobiological mechanisms of adenosine and P1 receptors in sleep, torpor, and hibernation

### 5.1 Increased levels of extracellular adenosine lead to drowsiness

Thanks to neurobiology and molecular biology advances, we are beginning to understand how sleep is initiated and maintained. Sustained wakefulness causes the body to produce and accumulate one or more endogenous somnogenic factors that induce sleep after reaching a certain threshold. The hypnotic effect of adenosine, an endogenous somnogenic factor, was discovered in 1954 (Feldberg and Sherwood, 1954). Typically, extracellular adenosine concentrations in the cerebral cortex and basal forebrain (BF) gradually increase during prolonged arousal, reaching a certain threshold that leads to drowsiness, while slowly decreasing during recovery sleep (Porkka-Heiskanen et al., 1997; Clasadonte et al., 2014; Huang et al., 2014; Tartar et al., 2021; Omond et al., 2022). Extracellular adenosine levels may be partially regulated by glutamatergic neurons (Peng et al., 2020; Sun and Tang, 2020). This is because activation of the glutamatergic BF neurons causes a large increase in extracellular adenosine, and specific ablation of glutamatergic BF neurons reduces the level of extracellular adenosine and significantly impairs sleep homeostasis regulation (Peng et al., 2020). Although adenosine is known to act on four evolutionarily conserved receptors, it is currently thought to regulate sleep-wake states by acting on the A_1_Rs and A_2A_Rs (Huang et al., 2014; Lazarus et al., 2019b).

### 5.2 Regulation of sleep homeostasis by A_1_Rs is brain region-dependent

A_1_Rs are required for normal sleep homeostasis because the conditional knockout of A_1_Rs in the CNS during sleep restriction results in a reduced rebound slow-wave activity response (Bjorness et al., 2009). Mainstream research suggests that activation of A_1_Rs promotes sleep, as A_1_Rs agonists increase sleep (Radulovacki et al., 1984; Benington et al., 1995), whereas A_1_Rs antagonists decrease sleep (Methippara et al., 2005; Thakkar et al., 2008). For example, when Oishi et al. (2008) injected the A_1_Rs-selective agonist N6-cyclopentyladenosine (CPA) into the rat tuberomammillary nucleus (TMN), this significantly increased NREM sleep. A_1_Rs may mediate sleep through three pathways (Lazarus et al., 2019b): 1) A_1_Rs promote sleep by inhibiting wake-promoting neurons. A_1_Rs are expressed in hypocretin/orexin neurons of the lateral hypothalamus (LH) and histaminergic neurons of the TMN, which are typical arousal centers. Activation of A_1_Rs inhibits excitatory neurotransmission, including cholinergic arousal systems in the brainstem (Rainnie et al., 1994) and BF (Alam et al., 1999; Thakkar et al., 2003), the hypocretin/orexin neurons in the LH (Thakkar et al., 2002; Liu and Gao, 2007), and histaminergic systems in the TMN (Oishi et al., 2008). 2) A_1_Rs promote sleep by disinhibiting sleep-active neurons in the ventrolateral preoptic nucleus (VLPO) and anterior hypothalamic area (Chamberlin et al., 2003; Morairty et al., 2004). 3) A_1_Rs mediate homeostatic sleep pressure based on astrocytic gliotransmission (Halassa et al., 2009).

Moreover, A1Rs do not appear to fully promote sleep because A_1_R knockout mice did not differ from wide-type mice in basal sleep amount and sleep-wake behavior after sleep deprivation (Stenberg et al., 2003). Infusion of CPA into the lateral ventricle of mice did not significantly alter NREM and REM sleep (Urade et al., 2003). However, microdialysis of the adenosine transporter inhibitor nitrobenzyl-thio-inosine (NBTIs) or A_1_R agonists into the lateral preoptic area (LPO) increased the amount of wakefulness in rats (Methippara et al., 2005). Thus, A_1_Rs may exert different sleep-wake effects by acting on different brain regions.

### 5.3 A_2A_Rs are important receptors that mediate the sleep-promoting effect of adenosine

A_2A_Rs are important targets in the regulation of sleep. A_2A_Rs mediate the effects of many sleep-promoting substances, such as ethanol and sake yeast (El Yacoubi et al., 2003; Nakamura et al., 2016; Fang et al., 2017; Nishimon et al., 2021). The selective A_2A_R agonist CGS21680 injected into the subarachnoid space adjacent to the BF and LPO of rats or the lateral ventricle of mice significantly increased NREM and REM sleep (Satoh et al., 1998; Scammell et al., 2001; Urade et al., 2003; Methippara et al., 2005). Immediately after the cessation of CGS21680 perfusion, there is a strong rebound in wakefulness (Gerashchenko et al., 2000). However, the sleep-promoting effect induced by CGS21680 was abolished entirely in A_2A_R knockout mice.

In addition, intraperitoneal administration of a positive A_2A_R allosteric modulator {3, 4-difluoro-2-[(2-fluoro-4-iodophenyl) amino] benzoic acid} in WT mice but not A_2A_R knockout mice enhanced A_2A_R signaling and promoted NREM sleep in a dose-dependent manner (Korkutata et al., 2019). Several studies suggested that A_2A_Rs mediated the sleep-regulating effects of prostaglandin D2 (PGD2). After administration of PGD2 or CGS21680 into the rostral BF, c-fos-positive cells were significantly increased in the VLPO, a sleep center, resulting in enhanced induction of NREM sleep, and in contrast, c-fos-positive neurons significantly decreased in the TMN of the posterior hypothalamus, a wake center (Satoh et al., 1999; Scammell et al., 2001). In *in-vivo* microdialysis experiments, infusion of CGS21680 into the BF dose-dependently decreased histamine release in the frontal cortex and medial preoptic area and increased GABA release in the TMN, but not in the frontal cortex (Hong et al., 2005). Furthermore, VLPO neurons have been divided into two types according to their different responses to serotonin and adenosine: Type-1 neurons were inhibited by serotonin, and type-2 neurons were excited. A_2A_R agonists excited postsynaptic type-2 neurons in the VLPO but not type-1 neurons. Type-2 neurons were involved in sleep initiation, whereas type-1 neurons may contribute to sleep consolidation because type-1 neurons were activated only when the inhibitory effects of the arousal system were absent (Gallopin et al., 2005). In addition to the VLPO, injection of CGS21680 into the rostral BF also increased c-fos expression in the shell of the NAc and the medial portion of the olfactory tubercle (OT) (Satoh et al., 1999; Scammell et al., 2001). Microinjection of CGS21680 into the NAc shell also induced sleep-promoting effects (Satoh et al., 1999). A_2A_Rs are highly expressed in the caudate putamen, NAc, and OT. Our recent series of studies have shown that activation of A_2A_R neurons in these nuclei can strongly promote sleep (Oishi et al., 2017; Yuan et al., 2017; Li et al., 2020). Activation of the A_2A_R neurons of the NAc core projecting to the ventral pallidum (VP) strongly induced NREM sleep. Conversely, inhibiting these neurons reduced sleep but did not affect the sleep homeostasis rebound (Oishi et al., 2017). Yuan et al. demonstrated the important role of the striatal A_2A_R neurons projecting to the external globus pallidus (GPe) parvalbumin (PV) neurons in sleep control. Chemogenetic inhibition of striatal A_2A_R neurons significantly decreased NREM sleep in the active period, which was mediated by the formation of inhibitory circuits between striatal A_2A_R neurons and GPe PV neurons (Yuan et al., 2017). The OT A_2A_R neurons project to the VP and LH *via* inhibitory innervations, and pharmacological or chemogenetic activation of OT A_2A_R neurons resulted in increased NREM sleep in mice (Li et al., 2020). Moreover, A_2A_Rs are co-localized with dopamine D_2_Rs in these nuclei (Missale et al., 1998). Our studies demonstrated that D_2_R-expressing neurons are essential for the induction and maintenance of wakefulness (Qu et al., 2008; Qiu et al., 2009; Qu et al., 2010; Liu Y. Y. et al., 2019; Yang et al., 2021). Thus, A_2A_Rs and D_2_Rs may jointly influence the sleep-wake cycle by balancing their activity.

Caffeine, unlike adenosine, is a wake-promoting substance abundant in refreshing beverages such as coffee and tea. Caffeine is an antagonist of A_1_Rs and A_2A_Rs, with similar affinity for both at low doses (Fredholm et al., 2001). Using A_1_R knockout and A_2A_R knockout mice, Huang et al. demonstrated that caffeine-induced wakefulness is dependent on A_2A_Rs, as caffeine dose-dependently increased wakefulness in both wild-type and A_1_R knockout but not A_2A_R knockout mice (Huang et al., 2005). Similarly, selective silencing of A_2A_Rs in the NAc shell inhibited caffeine-induced wakefulness (Lazarus et al., 2011).

**FIGURE 2:**
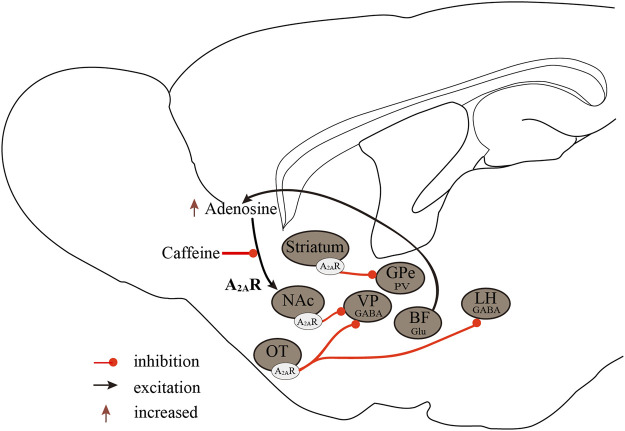
Neurobiological mechanisms of the A_2A_Rs regulate sleep-wake states. A_2A_Rs are important targets in sleep regulation, promoting sleep by inhibiting major arousal systems. Activation of A_2A_R neurons in the NAc core, striatum, and OT promotes sleep, with A_2A_Rs neurons in the NAc core projecting to the VP, striatal A_2A_R neurons, and GPe PV neurons forming inhibitory circuits, and OT A_2A_R neurons projecting to the VP and LH. Furthermore, BF glutamatergic neurons may regulate extracellular adenosine levels, and A_2A_Rs rather than A_1_Rs mediate the wake-promoting effects of caffeine. A_2A_Rs, adenosine A_2A_ receptors; A_1_Rs, adenosine A_1_ receptors; NAc, nucleus accumbens; VP, ventral pallidum; GPe, external globus pallidus; OT, olfactory tubercle; LH, lateral hypothalamus; Glu, glutamic acid; GABA, γ-aminobutyric acid.

In conclusion, the regulatory effect of A_1_Rs on sleep-wake regulation is brain region-dependent. The excitation of A_1_Rs in wake-promoting nuclei induces sleep and, conversely, causes arousal on sleep-promoting neurons. The A_2A_Rs are the major sleep-regulating receptors that mediate the wake-promoting effects of caffeine, and activation of A_2A_Rs promotes sleep by inhibiting major arousal systems (Figure 2).

### 5.4 Adenosine A_1_Rs and A_3_Rs play important roles in torpor

Adenosine may play a key role in torpor, as pyruvate induces torpor in obese mice based on adenosine signaling (Soto et al., 2018). In mice lacking all four adenosine receptors, adenosine does not cause hypothermia, bradycardia, or hypotension typical of the torpor state (Xiao et al., 2019). Peripheral or central infusion of adenosine or AMP results in a decrease in metabolic rate and body temperature similar to that observed in natural torpor, even in rats that do not naturally enter torpor (Swoap et al., 2007; Jinka et al., 2011; Iliff and Swoap, 2012; Olson et al., 2013; Tupone et al., 2013; Carlin et al., 2017; Vicent et al., 2017). Furthermore, the administration of A_1_R or A_3_R agonists to mice induces several features of daily torpor, including hypothermia (Anderson et al., 1994; Iliff and Swoap, 2012; Carlin et al., 2017; Swoap, 2017; Vicent et al., 2017), whereas A_2A_Rs and A_2B_Rs agonists do not (Anderson et al., 1994).

Currently, there are three ways to mimic the induction of torpor: 1) inhibition of the raphe pallidus (rPA) neurons in the brainstem (Cerri et al., 2021); 2) activation of A_1_Rs or A_3_Rs in the brain; 3) activation of glutamatergic Adcyap1+ neurons in the hypothalamus (Hrvatin et al., 2020). Here, we will discuss the induction of synthetic torpor by controlling A_1_Rs and A_3_Rs through pharmacological experiments. Although neither A_1_Rs nor A_3A_Rs are required for fasting-induced torpor (Carlin et al., 2017), administration of A_1_R or A_3_R agonists such as N6-cyclohexyladenosine (CHA) induces torpor-like states in some animals (Jinka et al., 2011; Olson et al., 2013; Tupone et al., 2013; Vicent et al., 2017; Frare et al., 2018), while antagonist administration prevents torpor or causes arousal from torpor during torpor phases (Jinka et al., 2011; Iliff and Swoap, 2012; Tamura et al., 2012). It is not yet certain whether adenosine action triggers the occurrence of natural torpor, but adenosine mediates at least some of the physiological features during torpor. For example, A_3_R stimulation leads to hypothermia *via* peripheral mast cell degranulation, histamine release, and activation of central histamine H_1_ receptors. However, A_1_R agonist-induced hypothermia occurs *via* central sites, and the rPA, nucleus of the solitary tract (NTS) and the hypothalamic-pituitary-thyroid axis gate appear to play a pivotal role (Tupone et al., 2013; Carlin et al., 2017; Frare et al., 2018).

In the future, further efforts should be made to confirm the role of adenosine in torpor and its possible neurobiological and molecular mechanisms. First, microdialysis experiments, adenosine probes, and chemogenetic and optogenetic techniques should be used to confirm whether there is an accumulation and dynamic change of adenosine concentration during the initiation and maintenance of torpor and to reveal the possible mechanisms.

### 5.5 Central activation of A_1_Rs is sufficient to induce and maintain a hibernation-like state

Seasonal changes in brain adenosine levels may contribute to an increase in A1R sensitivity leading to the onset of hibernation (Frare and Drew, 2021). Although the mechanisms controlling hibernation are currently unclear, activation of A_1_Rs signaling in the CNS appears to be required for the onset of this phenomenon, as activation of the A_1_Rs in the CNS can induce hibernation or some hibernation-like states in obligate, facultative, or non-hibernating animals (Drew et al., 2017; Shimaoka et al., 2018; Frare and Drew, 2021). In addition, Shimaoka et al. (2018) activated central A_1_Rs in rats, a non-hibernating animal, which induced a hypothermia response similar to hibernation.

It is worth noting that activation of A_1_Rs maintains core body temperature at a low level. In hibernators, core body temperature and metabolic rate reduction occur before hibernation, which may be the key to the A_1_R-mediated hibernation (Barros et al., 2006). A_1_Rs are highly expressed throughout the CNS, including the NTS. The NTS is the center that controls cardiovascular, respiratory, and metabolic functions, and the NTS neurons are responsible for the integration of central and peripheral signals related to energy expenditure-related (Barros et al., 2006). A_1_Rs act as inhibitory receptors whose activation prevents the release of GABA to the NTS neurons that inhibit thermogenesis (Cao et al., 2010). Furthermore, the administration of CHA to the arctic ground squirrel increased c-fos expression in the NTS in both summer and winter (Frare et al., 2019). After the microinjection of CHA into the NTS, it inhibited brown adipose tissue (BAT) thermogenesis and shivering responses. In contrast, inhibition of A_1_Rs counteracted BAT thermogenesis induced by intracerebroventricular injection of CHA (Tupone et al., 2013). In addition to inhibiting BAT thermogenesis, activation of A_1_Rs in the NTS increases vasopressin secretion, which constricts blood vessels, including skin vessels, thereby increasing arterial blood pressure (McClure et al., 2005; McClure et al., 2011) and causing bradycardia, one of the initial physiological features of natural hibernation (Jinka, 2012). The rPA, the median preoptic area (MnPO) and the supraoptic nucleus (SON) also appear to mediate the effect of A_1_Rs in BAT thermogenic, as the rPA and MnPO c-fos expression is lower in winter than in summer after CHA administration, and inhibition of rPA neurons produces hypothermia, however the SON is related to the seasonal increase in vasoconstriction (Cerri et al., 2013; Frare et al., 2019). Therefore, A_1_Rs could mediate hypothermia similar to hibernation by inhibiting BAT thermogenesis *via* the NTS and rPA or by inhibiting cardiovascular function. In addition, as previously mentioned, in contrast to sleep, EEG amplitudes are significantly reduced during hibernation (Golanov and Reis, 2001; Magdaleno-Madrigal et al., 2010). Central activation of A_1_Rs synchronized the EEG, whereas activation in the thalamus significantly reduced EEG amplitude (Saper et al., 2005). After central administration of CHA in rats, the EEG amplitude was greatly reduced, the delta wave amplitude was significantly reduced, and the theta wave almost disappeared (Tupone et al., 2013). Thus, the change in EEG amplitude may be another way A_1_Rs mediate hibernation.

As with torpor, it is currently unclear whether adenosine accumulation is necessary for the initiation of hibernation, so further efforts are needed to address these scientific questions.

## 6 Conclusion and future perspective

**FIGURE 3:**
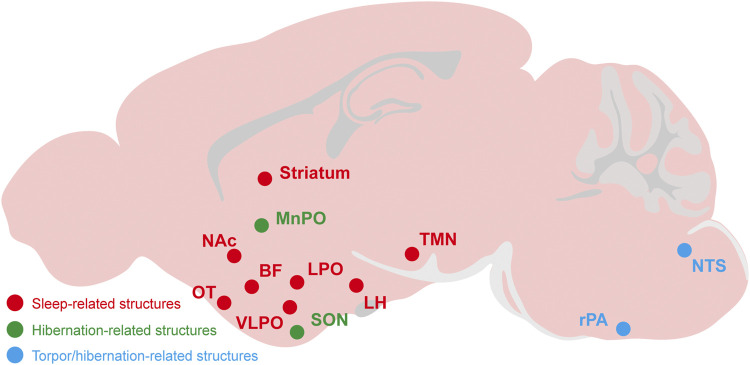
The relevant brain regions about adenosine and P1 receptors mediate sleep, torpor, and hibernation. The CNS adenosine and P1 receptors are important for the regulation of sleep-wake, torpor and hibernation. The roles and mechanisms of several brain regions and nuclei have been gradually revealed, such as the A_2A_Rs-expressing neurons in the NAc, striatum, OT and other structures have a significant effect on sleep-wake regulation. The NTS and rPA may be the key brain regions of adenosine and P1 receptors mediating torpor and hibernation. NAc, nucleus accumbens; OT, olfactory tubercle; LH, lateral hypothalamus; BF, basal forebrainvlpo; VLPO, ventrolateral preoptic nucleus; LPO, lateral preoptic area; MnPO, median preoptic area; SON, supraoptic nucleus; TMN, tuberomammillary nucleus; rPA, raphe pallidus; NTS, nucleus tractus solitarius.

In this review, we summarize the roles and neurobiological mechanisms of adenosine and its receptors in sleep-wake regulation, torpor, and hibernation (Table 2, Figure 3). The first step toward translating adenosine and P1 receptors into targets for medical applications is to understand their roles and mechanisms underlying these states of diminished metabolism and body temperature. We now know that A_1_Rs and A_2A_Rs jointly mediate sleep-wake regulation (Huang et al., 2014; Lazarus et al., 2019b), that activation of A_1_Rs and A_3_Rs is important for torpor (Carlin et al., 2017) and that hibernation requires A_1_Rs rather than other adenosine receptors (Shimaoka et al., 2018; Frare and Drew, 2021).

**TABLE 2:** Roles of adenosine receptors in sleep, torpor, and hibernation.

	Sleep	Torpor	Hibernation	References
Adenosine accumulation	Yes	Unknow	Unknow	Porkka-Heiskanen et al. (1997), Clasadonte et al. (2014), Huang et al. (2014), Tartar et al. (2021), Omond et al. (2022)
Key receptors	A_1_Rs, A_2A_Rs	A_1_Rs, A_3_Rs	A_1_Rs	
Related brain regions	TMN, LH, Brain stem, BF, VLPO, LPO, NAc, OT, Striatum	NTS, rPA, hypothalamus	NTS, rPA, MnPO, SON, thalamus	Huang et al. (2014), Yuan et al. (2017), Oishi et al. (2017), Shimaoka et al. (2018), Silvani et al., 2018, Li et al., 2020
Roles of adenosine receptors	A_1_R-mediated sleep-wake effects are brain region-dependent; A_2A_Rs promote sleep by inhibiting arousal systems	Activation of A_1_Rs or A_3_Rs mimic the induction of torpor	A_1_Rs may mediate hibernation *via* regulating core body temperature	Huang et al. (2014), Drew et al. (2017), Silvani et al. (2018), Lazarus et al. (2019a)

It is worth noting that the adenosine system is also altered in various sleep disorders, for example, sleeping sickness and chronic insomnia disorder (Rijo-Ferreira et al., 2020; Ren et al., 2021). Some agonists, antagonists, or allosteric modulators targeting adenosine receptors have the potential to be used for treating sleep disorders (Jenner et al., 2020; Korkutata et al., 2022) or inducing synthetic torpor or hibernation for therapeutic hypothermia, organ preservation, space exploration or longevity promotion (Jinka et al., 2015; Cerri, 2017; Sisa et al., 2017; Hadj-Moussa and Storey, 2019; Al-Attar and Storey, 2020; Cerri et al., 2021), showing that the pharmacological importance of targeting adenosine receptors in the future. However, much work remains to be done because small-molecule drugs targeting adenosine receptors have side effects (Korkutata et al., 2022) and can only mimic some physiological properties of torpor or hibernation by activating adenosine receptors, which is different from natural torpor or hibernation (Swoap, 2017; Vicent et al., 2017). Therefore, it is necessary to explore further the roles and mechanisms of adenosine and its receptors in sleep, torpor, and hibernation and gain more adenosine receptor modulators by structure- and function-based drug discovery. It is important to investigate the neural networks and molecular mechanisms that sleep torpor and hibernation have in common. The first step in conducting these studies is to confirm adenosine accumulation before torpor or hibernation and the dynamic changes in adenosine concentrations during torpor or hibernation using available technologies such as microdialysis, adenosine probes, and chemogenetic and optogenetic methods. Subsequently, several key technologies, from conditional knockout mice based on Cre/lox technology and RNA interference to modulation of neuronal activity with genetic or pharmacological techniques, can be used to confirm neuronal networks of sleep, torpor, and hibernation.

## Glossary

**A**_**1**_ **Rs**: adenosine A_1_ receptors
**A**_**2A**_ **Rs**: adenosine A_2A_ receptors
**A**_**2B**_ **Rs**: adenosine A_2B_ receptors
**A**_**3**_ **Rs**: adenosine A_3_ receptors
AC: adenylate cyclase
ADK: adenosine kinase
ADP: adenosine diphosphate
AMP: adenosine monophosphate
ATP: adenosine triphosphate
BAT: brown adipose tissue
BF: basal forebrain
BMR: basal metabolic rate
BP: blood pressure
cAMP: cyclic adenosine-3,5 monophosphate
CHA: N6-cyclohexyladenosine
CNS: central nervous system
CPA: N6-cyclopentyladenosine
EEG: electroencephalographic
EMG: electromyography
E-NTPDase: ecto-nucleoside triphosphate diphosphohydrolase
Gi: inhibitory adenylate cyclase G protein
GPCR: G protein coupled receptor
GPe: external globus pallidus
Gs: stimulating adenylate cyclase G protein
HP: heart period
HR: heart rate
KATP: ATP sensitive potassium channel
LH: lateral hypothalamus
MR: metabolic rates
NAc: nucleus accumbens
NBTIs: nitrobenzyl-thio-inosine
NREM: non-rapid eye movement
NTS: nucleus tractus solitarius
OT: olfactory tubercle
PAM: positive allosteric modulator
PLC: phospholipase C
PV: parvalbumin
REM: rapid eye movement
rPA: raphe pallidus
MnPO: median preoptic
SON: supraoptic
SAH: S-adenosylhomocysteine
SAHH: S-adenosylhomocysteine hydrolase
SAM: S-adenosylmethionine
SWS: slow-wave sleep
Ta: ambient temperature
**T**_**b**_: body temperature
TMN: tuberomammillary nucleus
VLPO: ventrolateral preoptic nucleus lateral preoptic area
LPO: lateral preoptic
VP: ventral pallidum γ-aminobutyric acid
GABA: γ-aminobutyric acid
5′-NT: 5′-nucleotidase
